# Targeted and untargeted detection of fentanyl analogues and their metabolites in hair by means of UHPLC-QTOF-HRMS

**DOI:** 10.1007/s00216-020-02994-x

**Published:** 2020-10-15

**Authors:** Alberto Salomone, Daniele Di Corcia, Pierre Negri, Maria Kolia, Eleonora Amante, Enrico Gerace, Marco Vincenti

**Affiliations:** 1 Centro Regionale Antidoping e di Tossicologia, Regione Gonzole 10/1, 10043 Orbassano, TO Italy; 2 Department of Chemistry, University of Turin, Via Pietro Giuria 5, 10125 Torino, Italy; 3 AB SCIEX, Redwood City, CA 01701 USA; 4 Department of Chemistry, University of Ioannina, Ioannina Campus, 1186, Ioannina, 45500 Greece

**Keywords:** Hair, NPS, QTOF, NSO, SWATH, Fentanyl, Untargeted

## Abstract

Detection of new psychoactive substances and synthetic opioids is generally performed by means of targeted methods in mass spectrometry, as they generally provide adequate sensitivity and specificity. Unfortunately, new and unexpected compounds are continuously introduced in the illegal market of abused drugs, preventing timely updating of the analytical procedures. Moreover, the investigation of biological matrices is influenced by metabolism and excretion, in turn affecting the chance of past intake detectability. In this scenario, new opportunities are offered by both the non-targeted approaches allowed by modern UHPLC-HRMS instrumentation and the investigation of hair as the matrix of choice to detect long-term exposure to toxicologically relevant substances. In this study, we present a comprehensive and validated workflow that combines the use of UHPLC-QTOF-HRMS instrumentation with a simple hair sample extraction procedure for the detection of a variety of fentanyl analogues and metabolites. A simultaneous targeted and untargeted analysis was applied to 100 real samples taken from opiates users. MS and MS/MS data were collected for each sample. Data acquisition included a TOF-MS high-resolution scan combined with TOF-MS/MS acquisition demonstrating considerable capability to detect expected and unexpected substances even at low concentration levels. The predominant diffusion of fentanyl was confirmed by its detection in 68 hair samples. Other prevalent analogues were furanylfentanyl (28 positive samples) and acetylfentanyl (14 positive samples). Carfentanil, methylfentanyl, and ocfentanil were not found in any of the analyzed samples. Furthermore, the retrospective data analysis based on untargeted acquisition allowed the identification of two fentanyl analogues, namely β-hydroxyfentanyl and methoxyacetylfentanyl, which were not originally included in the panel of targeted analytes.

**Electronic supplementary material:**

The online version of this article (10.1007/s00216-020-02994-x) contains supplementary material, which is available to authorized users.

## Introduction

In the last decade, the general situation and world distribution of drugs of abuse has evolved dramatically with the emergence of a large variety of new psychoactive substances (NPS). In particular, the pattern of abused synthetic opioids progressed from the over-prescription of legal analgesic drugs such as hydrocodone, oxycodone, and fentanyl, to the clandestine synthesis of new fentanyl derivatives specifically produced for the illegal market [1, 2]. Fentanyl and its analogues are considered particularly risky for their extreme pharmacological effects, as the large and ever-increasing outburst of lethal overdose cases in the USA clearly evidences [3]. These fatal overdoses may either stem from the use of fentanyl and analogues as cutting/adulterant agents or simply as substitutes for heroin. The easy accessibility of these powerful substances poses a major safety concern for both drug abusers and law enforcement officials exposed to the seized materials. The timely detection of individual exposure to fentanyl and its analogues, potentially lethal even at low dosage, represents a challenging objective for both their typically minuscule concentration in body fluids and their chemical variability associated to minor structural changes of the parent drug. Detection of fentanyl analogues can be performed in many biological matrices, including urine, blood, saliva, and hair. Unlike blood and urine, hair analysis is increasingly used to detect long-term exposure to toxicologically relevant substances, as it offers a wide diagnostic window basically dependent on the hair length [4]. Hair analysis has been repeatedly used to ascertain past exposure and prevalence of different novel synthetic opioids (NSO) in the consumer’s population [5–14]. The analytical methods currently available are generally based on the targeted detection of a limited and well-defined list of compounds to monitor, usually chosen on the base of the national or international reports, or alerts from national warning systems [15, 16]. One of the challenges in the development of validated methods for the analysis and identification of NSO within a rapidly changing and dynamic market is that analytical reference materials may not be commercially available or require long time to be synthesized. Therefore, most toxicological laboratories are stimulated to create up-to-date targeted methods, capable of detecting dozens of compounds whose list is constantly renovated. Nevertheless, effective approaches for NSO screening may benefit from recent technological developments of analytical instrumentation and methods [17]. In particular, untargeted screening methods are devoted to a comprehensive investigation of the tested samples, by (i) looking for compounds structurally similar to the targeted drugs, (ii) proposing their identities, and (iii) confirming the new findings and add them to the target list. Preliminary HRMS-based approaches have been recently proposed, in order to screen for different classes of drugs in hair [8], for fentanyl analogues in blood [18], or for emerging synthetic cannabinoids [19]. In our study, we aimed to develop, validate, and apply a new analytical method based on a simultaneous targeted and untargeted approach. The comprehensive workflow combined the use of a UHPLC-QTOF-HRMS system, with a simple extraction procedure for specific and sensitive detection of fentanyl analogues in hair. The untargeted investigation based on a retrospective data analysis proved qualified to perform untargeted screening without the need of analytical standards.

## Experimental

### Reagents and standards

Reagents and standards of furanylfentanyl, 4-fluorobutyrfentanyl, acrylfentanyl, butyrylfentanyl, and 4-anilino-N-phenethyl-piperidine (4-ANPP) were produced by Chiron (Trondheim, Norway). Acetylfentanyl and carfentanil were obtained by Toronto Research Chemicals (North York, Canada) while ocfentanil and norfentanyl were purchased from Sigma-Aldrich (Milan, Italy). Cyclopropylfentanyl, α-methylfentanyl, and β-hydroxyfentanyl were provided by National Institute of Health (ISS). Fentanyl and the deuterated internal standards (norfentanyl-*d*_*5*_, fentanyl-*d*_*5*_) were produced by Cerilliant (Round Rock, Texas, USA). All other chemicals were purchased from Sigma-Aldrich (Milan, Italy). Ultra-pure water was obtained using a Milli-Q® UF-Plus apparatus (Millipore, Bedford, MA, USA). All stock standard solutions were prepared in methanol at 1 mg/mL and stored at − 20 °C until used. Working solutions were prepared at the final concentration of 1000 ng/mL by dilution with methanol.

### Sample preparation

About 50 mg of head hair were twice-washed with dichloromethane and then methanol (1 mL of solvent, vortex mixed for 3 min). The solvent washes were removed following each vortex mixing steps. Following the washing steps, hair was dried at room temperature using a gentle nitrogen flow and subsequently grinded with a ball mill (Precellys 24, Bertin Instruments, Montigny-le-Bretonneux, France). The resulting hair sample was spiked with 2.5 μL of an internal standard mixture yielding a final concentration of 50 pg/mg. One milliliter of HPLC-grade methanol was added and the mixture was incubated at 55 °C for 15 h without stirring. Following the incubation step, 100 μL of the organic phase was transferred in a UHPLC vial and an aliquot of 3 μL was directly injected into the UHPLC-HRMS/MS system. Whenever the real sample concentrations were found to exceed the highest calibration point, the final extracts were diluted with methanol and re-injected into the system.

### Instrumentation

UHPLC separation was performed on a Phenomenex Kinetex C18 column (100 × 2.1 mm, 1.7 μm, 00D-4475-AN) at 45 °C on the SCIEX ExionLC™ AC system. Mobile phases consisted of water (A) and acetonitrile (B), both with 5 mM of formic acid. The LC flow rate was 0.5 mL and the mobile phase eluted under the following linear gradient conditions: (A:B, v:v) isocratic elution at 95:5 for 0.5 min, from 95:5 to 50:50 in 4.5 min, isocratic elution at 50:50 for 0.5 min, and final re-equilibration for 2.5 min to the initial condition before each injection. Total run time was 7 min.

All analyses were performed using a quadrupole time-of-flight SCIEX X500R QTOF mass spectrometer (Sciex, Darmstadt, Germany) equipped with a Turbo V™ ion source operating in electrospray positive-ion mode. MS and MS/MS data were collected for each sample using SWATH™ Acquisition mode. Data acquisition included a preliminary TOF-MS high-resolution scan followed by SWATH™ Acquisition using variable window setup (12 windows covering mass range from 230 to 450 *m/z* at 0.02 resolving power), resulting in a final cycle time of 0.555 s. Data were acquired using SCIEX OS 1.5 Software. The full list of the MS/MS transitions for the analytes and internal standards is presented in Table 1.

**Table 1:** Full list of the MS/MS transitions for analytes and internal standards

	Name	Elemental composition	Precursor ion	Precursor (Q1) calculated mass (Da)	Fragment (Q3) calculated mass (Da)
1	Norfentanyl	C_14_H_20_N_2_O	[M + H]^+^	233.1648	84.0808
2	Acetylfentanyl	C_21_H_26_N_2_O	[M + H]^+^	323.2118	188.1434
3	Ocfentanil	C_22_H_27_FN_2_O_2_	[M + H]^+^	371.2129	188.1434
4	Acrylfentanyl	C_22_H_26_N_2_O	[M + H]^+^	335.2118	188.1434
5	4-ANPP	C_19_H_24_N_2_	[M + H]^+^	281.2012	188.1434
6	Fentanyl	C_22_H_28_N_2_O	[M + H]^+^	337.2274	188.1434
7	Furanylfentanyl	C_24_H_26_N_2_O_2_	[M + H]^+^	375.2067	188.1434
8	α-Methylfentanyl	C_23_H_30_N_2_O	[M + H]^+^	351.2431	202.159
9	Cyclopropylfentanyl	C_23_H_28_N_2_O	[M + H]^+^	349.2274	188.1434
10	Carfentanil	C_24_H_30_N_2_O_3_	[M + H]^+^	395.2329	335.2118
11	Butyrfentanyl	C_23_H_30_N_2_O	[M + H]^+^	351.2431	188.1434
12	4-Fluorobutyrfentanyl	C_23_H_29_FN_2_O	[M + H]^+^	369.2337	188.1434
IS1	Norfentanyl-*d*_*5*_	C_14_H_15_^2^H_5_N_2_O	[M + H]^+^	238.1962	–
IS2	Fentanyl-*d*_*5*_	C_22_H_23_^2^H_5_N_2_O	[M + H]^+^	342.2588	188.1434

### Data analysis and processing

Data processing was performed using SCIEX OS 1.5 Software for positive analyte identification based on confidence criteria. The four main confidence criteria used include precursor and fragment mass error ± 5 ppm and library hit score (L). Subsequently, a combined score (C) was calculated based on the third confidence categories (MRIL) with custom weightings.

A data processing method was developed to review the SWATH ™ Acquisition data. While data acquisition was set in the non-targeted mode, data processing was organized with targeted approach, using a list of 12 targeted analytes and 2 internal standards to initially screen the dataset. Then, further screening of related and potentially interesting compounds not initially targeted was achieved by querying the software to look for their protonated exact mass. The full list of these molecules is shown in Table S1 (see Electronic Supplementary Material, ESM). When an unknown peak was observed, additional software processing and functional relationship search was activated to determine the potential candidate formula and structure. The workflow utilizes the experimentally determined high-resolution and accurate mass of the detected peak and the Formula Finder feature to generate candidate empirical formulae for the corresponding molecule. These candidate formulae were coupled with MS/MS fragmentation spectra and matched with the extensive ChemSpider database to verify whether the predicted in silico fragmentation pattern of the candidate structures corresponded to experimental MS/MS spectra. Once the exact mass and isotopic profile of the unknown molecule is selected, the software links these features to the ChemSpider site which is a free chemical structure database providing fast text and structure search access to over 67 million structures from hundreds of data sources. ChemSpider can generate candidate structures for each formula with a matching of HRMS/MS spectra to predicted fragment ions [20].

### Real samples

Real hair samples were collected in the USA between November 2016 and August 2018. All samples selected for the present study had previously tested positive to heroin or its metabolites by means of a targeted LC-MS/MS method. A total of 100 samples was analyzed. All samples were analyzed in their entire length (range 1–20 cm, mean value 4.0 cm), following international recommendations [21]. In order to nullify the risk related to data sharing and to safeguard the privacy of sample donors, all samples were made anonymous by alphanumeric codes and used only in our laboratory. The risk of re-identification was also nullified. Furthermore, donors had given informed consent to be tested for drugs of abuse. The aim of our study was indeed to identify the intake of a certain drug, albeit by means of untargeted approach. No other information was obtained from the reuse of these samples, except the possible exposure to a certain drug, as already verified by targeted analyses.

### Validation

The calibration process was conducted with an optimized procedure, requiring the preparation of three replicates of the calibration curves for the targeted compounds in three different days for a total of nine calibration curves [22, 23]. Several validation parameters were determined from these data, including linearity range and calibration model, selectivity, specificity, limit of detection (LOD), limit of quantification (LOQ), trueness, intra and inter-assay precision, and repeatability. The linearity was evaluated within the concentration range of 2.0–100 pg/mg. The best calibration model was determined using the RStudio routine developed by Desharnais et al. [24, 25], which included the study of homo- vs. heteroscedasticity (and the correction of it by means of the proper weight) and order of the calibration model (linear or quadratic). The LOD and LOQ were estimated by means of the Hubaux-Vos’ algorithm [26] applied in the linear dynamic range and corrected for the heteroscedasticity weights [22].

To determine selectivity and specificity, the signal-to-noise ratio (S/N) was measured on the selected ion chromatograms at the expected retention times for all the analytes of interest. The presence of interfering peaks around the retention time of the analytes was identified by S/N values higher than 3. Intra- and inter-day precision and accuracy were evaluated using two dedicated back-calculation approaches, described elsewhere [22]. Optimal percent coefficient of variation (CV%) and percent bias were expected to lie within ± 15%, while results within ± 25% were still considered satisfactory. Retention time (RT) repeatability was verified on 30 real hair samples together with blank hair samples spiked at different concentration levels. RT deviations below 1% from calibrators and controls were considered acceptable. Carry-over effect was evaluated by injecting one blank extracts after the highest point of each calibration curve: if the S/N ratio was lower than 3 for each ion chromatogram, the carry-over effect was considered negligible. Since the matrix effect is likely to have a more critical impact on quantitative results when the concentrations are in the low range of calibration, this parameter was estimated only at the 2 pg/mg concentration levels by comparing the experimental results obtained from five blank hair samples and solutions of pure methanol, both spiked after the extraction step. The matrix effect for each target analyte was expressed as the percentage ratio between the two measured concentrations.

## Results and discussion

**Fig. 1:**
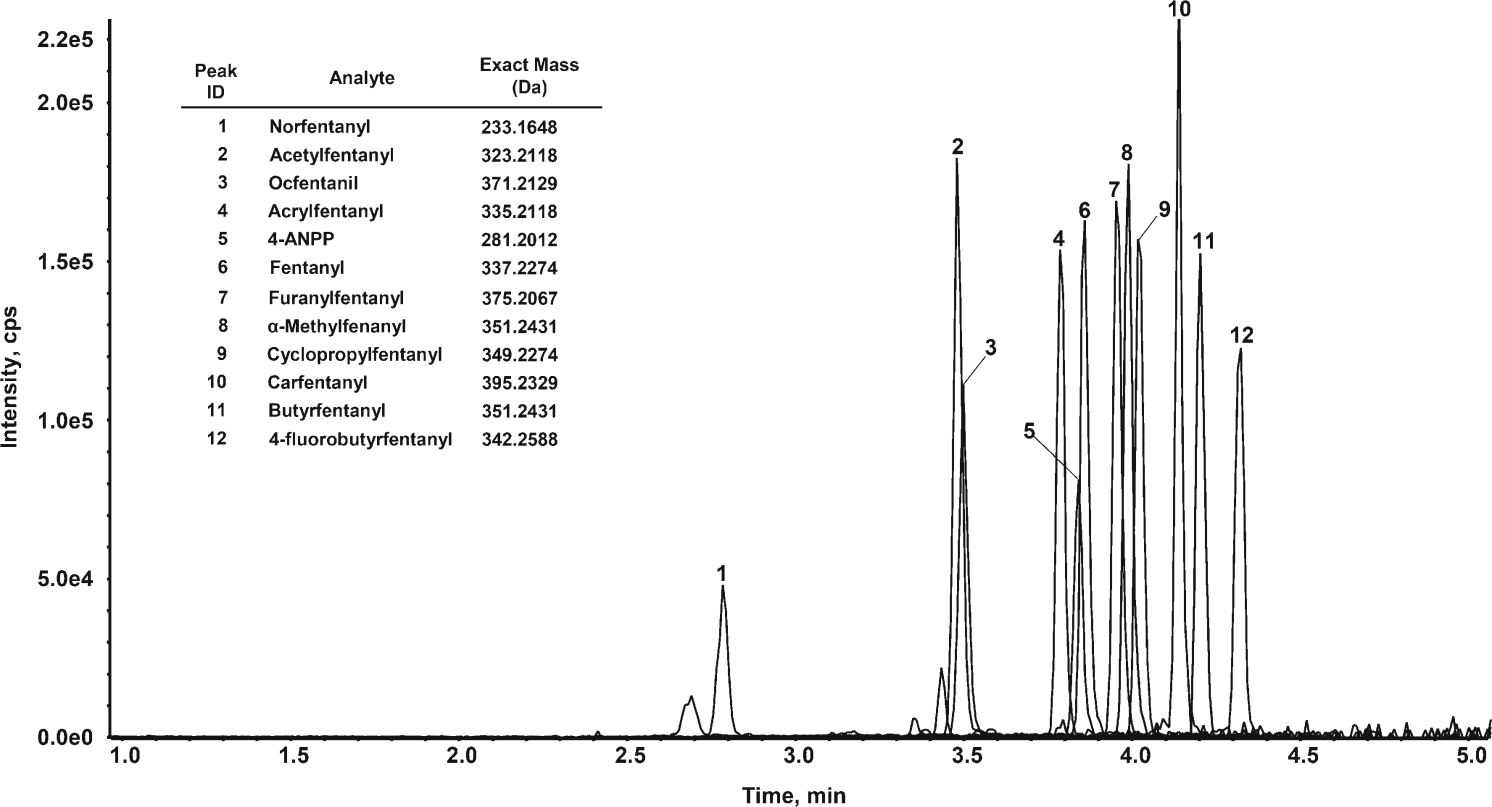
TIC chromatogram recorded from a blank hair spiked with all analytes at 100 pg/mg concentration. The order of elution is as follows: (1) norfentanyl, (2) acetylfentanyl, (3) ocfentanil, (4) acrylfentanyl, (5) 4-ANPP, (6) fentanyl, (7) furanylfentanyl, (8) α-methylfentanyl, (9) cyclopropylfentanyl, (10) carfentanil, (11) butyrfentanyl, (12) 4-fluorobutyrfentanyl

The optimized choice of LC conditions including column selection and mobile phase composition and gradient resulted in satisfactory separation of all targeted analytes, comprising the closely related fentanyl analog compounds present in the stock standard solution mixture. The whole chromatographic run, comprehensive of the time required for column re-equilibration before the following injection, was completed in 5 min. Retention times ranged between 2.76 min (norfentanyl) and 4.28 min (4-fluorobutyrfentanyl). Figure 1 shows the TIC chromatogram recorded from a blank hair spiked with all analytes at 100 pg/mg concentration.

### Validation

Table 2 reports a summary of the observed results including the range of calibration, calibration curve equations, and LODs and LOQs values. For all the analytes, the calibration data points proved to have heteroscedastic distribution suggesting the homogeneous use of a 1/x^2^ weighting factor. The calibration curves for all analytes proved linear within the calibration range, after lack-of-fit and Mandel testing.

**Table 2:** Validated parameters for the targeted screening

	Compound	Internal standard	Calibration range (pg/mg)	Weight	Calibration curve	LOD (pg/mg)	LOQ (pg/mg)
1	Norfentanyl	Norfentanyl-*d*_*5*_	2.0–100	1/x^2^	3.31x + 0.04	1.2	2.4
2	Acetylfentanyl	Fentanyl-*d*_*5*_	2.0–100	1/x^2^	4.46x + 0.03	0.6	1.2
3	Ocfentanil	Fentanyl-*d*_*5*_	2.0–100	1/x^2^	3.57x + 0.01	0.4	0.8
4	Acrylfentanyl	Fentanyl-*d*_*5*_	2.0–100	1/x^2^	3.48x + 0.01	0.6	1.2
5	4-ANPP	Fentanyl-*d*_*5*_	2.0–100	1/x^2^	3.78x + 0.01	0.7	1.4
6	Fentanyl	Fentanyl-*d*_*5*_	2.0–100	1/x^2^	4.58x + 0.02	0.6	1.2
7	Furanylfentanyl	Fentanyl-*d*_*5*_	2.0–100	1/x^2^	5.79x + 0.02	0.6	1.2
8	α-Methylfentanyl	Fentanyl-*d*_*5*_	2.0–100	1/x^2^	2.32x −0.002	0.5	1.0
9	Cyclopropylfentanyl	Fentanyl-*d*_*5*_	2.0–100	1/x^2^	3.57x + 0.02	0.7	1.4
10	Carfentanil	Fentanyl-*d*_*5*_	2.0–100	1/x^2^	3.26x + 0.002	0.8	1.6
11	Butyrfentanyl	Fentanyl-*d*_*5*_	2.0–100	1/x^2^	4.10x + 0.02	0.6	1.2
12	4-Fluorobutyrfentanyl	Fentanyl-*d*_*5*_	2.0–100	1/x^2^	3.49x + 0.02	0.2	0.4

Retention time precision, selectivity, and specificity proved satisfactory, and no interfering signals were detected at the retention times of the target analytes. Inter-day and intra-day precision (expressed as percent variation coefficient, CV%) and accuracy (expressed as bias%) were found to be below 25% and 20%, respectively. The assay showed remarkable reproducibility for concentrations ranging over three orders of magnitude, proving the robustness of the overall workflow. Limits of detection (LOD) in matrix were found to be in the sub pg/mg range for most of the target analytes used in this study (see Table 2). Lastly, the absence of any carry-over effect was observed. The validation results not reported in Table 2 are summarized in Table S2 (see ESM). The real hair matrix effect appeared to be significant for some of the analytes tested. However, the matrix effect is expected to be partly compensated by a well-matched internal standard, i.e., the isotopically labeled analyte, whenever possible, or the one having similar retention time and structural features.

### Targeted analysis of real samples

**Fig. 2:**
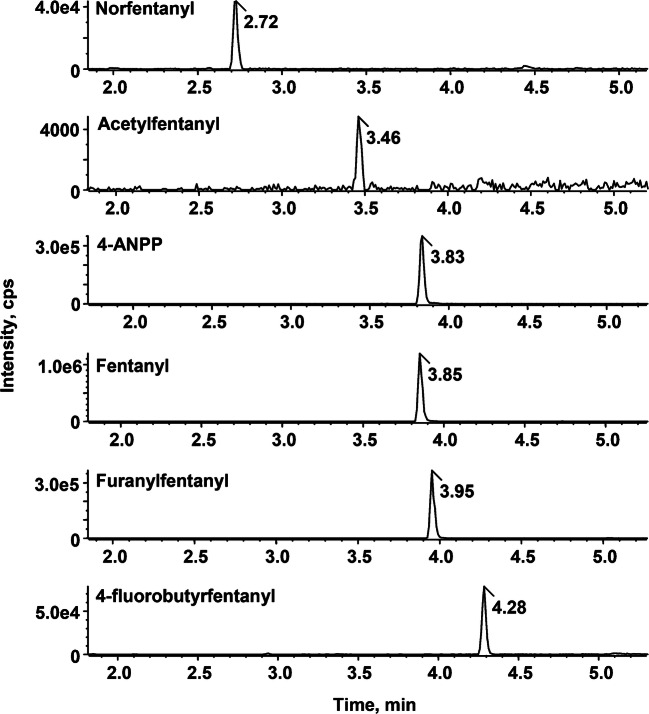
Extracted ion chromatograms (mass tolerance ± 5 ppm) from a real sample positive to fentanyl (420 pg/mg), norfentanyl (18 pg/mg), 4-ANPP (230 pg/mg), acetylfentanyl (2.2 pg/mg), furanylfentanyl (120 pg/mg), and 4-fluorobutyrfentanyl (36 pg/mg)

The large diffusion all over the USA of fentanyl (and its analogues), either as substitute or cutting material of heroin, makes hair samples collected from habitual opiates users the ideal real matrices to test the robustness of the workflow developed in the present study. Unlike the selected reaction monitoring procedures commonly used in triple-quadrupole instruments to detect the targeted analytes, the SWATH ™ Acquisition was chosen to produce complete high-resolution MS/MS spectra, enabling fully reliable identification based on several highly specific accurate mass fragment ions and library database matching. In practice, the SCIEX OS Software provides a centralized result grid that allows simultaneous quantification and library matching, displaying at the same time and within a single window the XIC, TOF-MS, and MS/MS spectra of the candidate analyte with library search match. In addition, retention time, mass, isotope ratio error, and mass spectral library search score are automatically calculated and visualized. Figure 2 shows the successful detection of fentanyl, acetylfentanyl furanylfentanyl, and 4-fluorobutyrfentanyl from one of the tested head hair samples at concentrations of 420, 2, 120, and 36 pg/mg, respectively, together with the metabolites norfentanyl and 4-ANPP, at concentration of 18 and 230 pg/mg, respectively. The library fit scores (> 99.0%) and the combined scores (> 90%) provided excellent confidence for the definitive detection of these NSOs.

Overall, 70 samples out of 100 tested positive to at least one target analyte among the ones listed in Table 1. Fentanyl was the predominant synthetic opioid, being present in 68 hair samples with concentrations in the 1.2–1400 pg/mg range. Other prevalent analogues were furanylfentanyl (28 positive samples, range 1.2–6300 pg/mg) and acetylfentanyl (14 positive samples, range 1.2–230 pg/mg). The metabolite norfentanyl was detected in 17 cases, with concentrations in the range 3.5–600 pg/mg, while the precursor/metabolite 4-ANPP was detected in 20 cases, with concentrations in the range 1.4–230 pg/mg. The complete panel of positive findings is reported in Table 3. Notably, carfentanil, methylfentanyl, and ocfentanil were not found in any of the analyzed samples. The possible reasons for the rare occurrence of samples testing positive to carfentanil are (1) the low prevalence at the time of the sample collection, (2) poor incorporation or low stability of carfentanil in the keratin matrix, and/or (3) insufficient sensitivity of the analytical method in relation to the low effective dosage. On the other hand, fentanyl itself is possibly potent enough to exclude the need of introducing further potentially lethal substances into the illegal market. Indeed, more investigation is still needed before a final interpretation is given for the low occurrence of carfentanil in hair testing [7].

**Table 3:** Summary of results obtained from 100 real hair samples

Target analyte	Number of positive samples	Range of concentrations (pg/mg)	Mean pg/mg	Median pg/mg
Fentanyl	68	LOQ–1400	93	17
Norfentanyl	17	3.5–600	69	8.4
Acetylfentanyl	14	LOQ–230	29	2.5
Furanylfentanyl	28	LOQ–6300	310	8.4
Acrylfentanyl	2	LOQ	–	–
4-Fluorobutyrfentanyl	6	5.2–180	69	24
Cyclopropylfentanyl	1	4.7	–	
Butyrfentanyl	1	54		
4-ANPP	20	1.4–230	22	4.1

### Retrospective untargeted analysis of real samples

A major motivation for the use of SWATH ™ Acquisition mode is the opportunity to detect relevant analytes not included in the list of expected substances. While the availability of a pure standard is necessary for accurate quantification, unexpected analytes can be identified with confidence when high-resolution TOF-MS and MS/MS, ad isotope distribution results are matched with library data. However, this opportunity represents a serious challenge in the case of fentanyl analogues and metabolites, because their extremely high biological activity is frequently consistent with low administered dosages, further reducing the already minimal amount of drug partitioned in the hair matrix. In the illegal market, the substances with the highest pharmacological potency and lowest dosages are particularly attractive to dealers.

The retrospective untargeted analysis of the real samples considered in this study allowed the identification of two fentanyl analogues which were not included in the panel of targeted analytes. In one sample, the occurrence of β-hydroxyfentanyl was proposed as the best match from ChemSpider, and afterwards confirmed by comparison with the analytical standard purposely acquired. β-Hydroxyfentanyl was originally sold as an illicit drug in the 1980s but its use has not been reported since that time [27]. Very recently, a case of toxicity from intentional therapeutic use of β-hydroxyfentanyl (possibly mistaken as fentanyl) was reported [28]. Furthermore, β-hydroxyfentanyl had been detected in biological fluids as a metabolic product after fentanyl administration [29, 30], but it has never been reported in hair before.

**Fig. 3:**
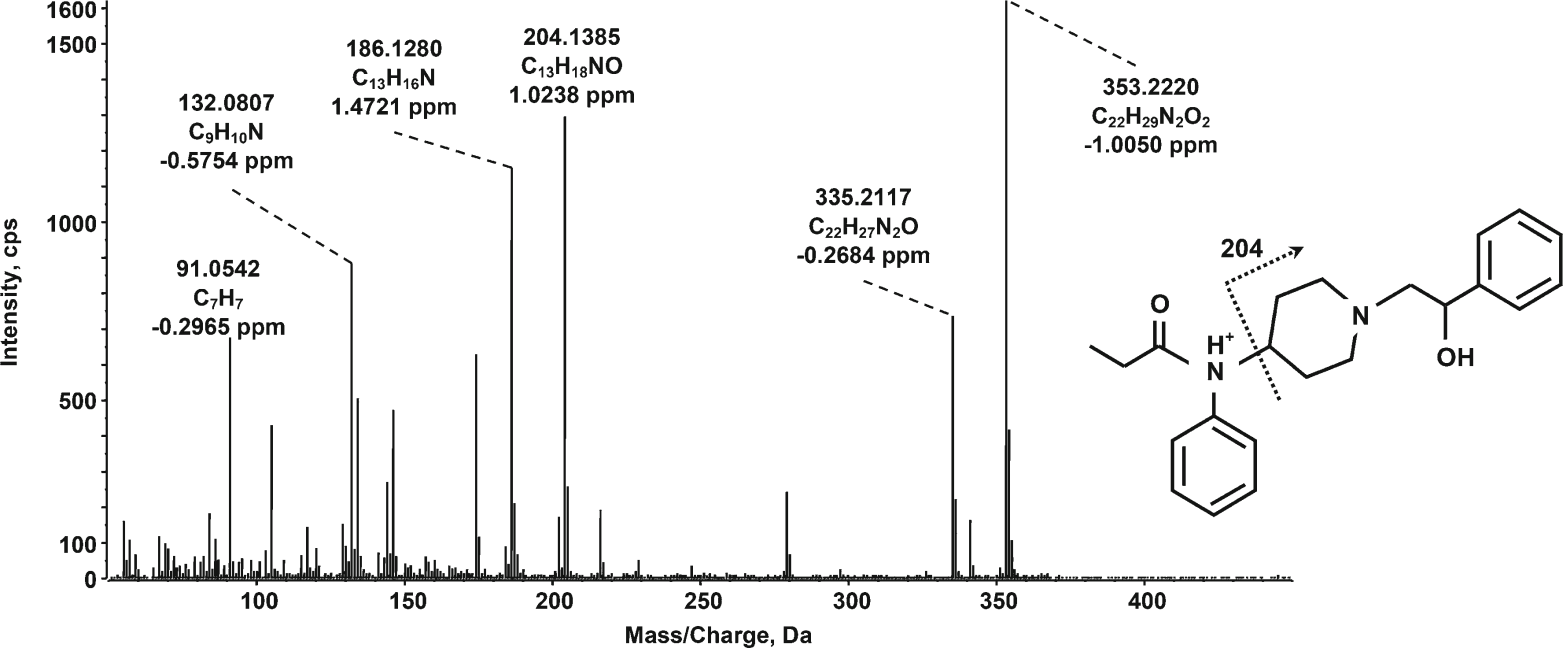
TOF-MS/MS spectrum and predominant fragmentation pattern of β-hydroxyfentanyl as observed in a real hair sample

The concurrent detection of metabolites of the taken substance in hair represents an essential step in analytical toxicology to sustain the hypothesis of deliberate abuse and exclude the alternative hypothesis of external contamination from the parent drug [4]. In this context, the identification of β-hydroxyfentanyl can open new opportunities to assist the result interpretation in hair analysis, even if further studies are needed to investigate its presence in a larger population of fentanyl-positive samples and to evaluate the typical concentration ratio between parent drug and β-hydroxyfentanyl. The proposed fragmentation scheme for β-hydroxyfentanyl is shown in Fig. 3. Major fragments include *m/z* 335 (corresponding to a water loss from the protonated molecular ion), *m/z* 204 (corresponding to the hydroxyphenethyl-piperidine moiety), the subsequent water loss (*m/z* 186) from *m/z* 204, and the tropylium cation at *m/z* 91 (formed from the phenethyl moiety). The fragment *m/z* 132 is likely to correspond to the N-propenyl-aniline ion structure.

**Fig. 4:**
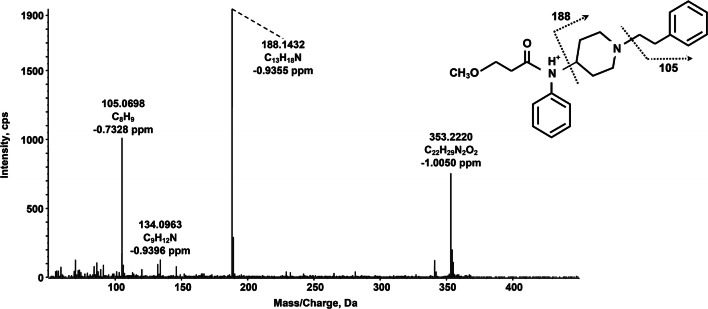
TOF-MS/MS spectrum and predominant fragmentation pattern of methoxyacetylfentanyl as observed in a real hair sample

In a different sample, the software attributed a chromatographic peak to the presence of methoxyacetylfentanyl. This compound is among the latest fentanyl analogues emerged onto the recreational drug scene, potentially being sold to unsuspecting users as a contaminant or substitute for heroin. As a new active substance, it has been linked to several intoxication cases, mostly lethal [31, 32]. To our knowledge, we present hereby the first tentative identification of this analyte in hair, based on literature data which should be confirmed as soon as a commercial reference standard will be available. The chemical formula of the methoxyacetylfentanyl protonated molecular ion was identified with an error of 0.6 ppm, while the proposed fragmentation pattern is shown in Fig. 4. The fragments at *m/z* 188 and 105 are typical for fentanyl and its analogues. In particular, the fragment at *m/z* 188 is consistent with the phenethyl-piperidine moiety, while the fragment at *m/z* 105 corresponds to the phenethyl ion.

## Conclusions

In the development of analytical methods, the prerequisite of multi-analyte protocols is that the list of the targeted substances includes all the molecules of interest expected to be possibly present in the screened samples. Unfortunately, the toxicological analyses dealing with the detection of NPS/NSO cannot be counted within such an ideal scenario. Quite often, new and unexpected compounds abruptly show up in the illegal market of drugs of abuse. While the existing targeted screening methods have proved remarkable sensitivity and specificity, their inability to detect new compounds which are not included in the panel of target analytes appears as a significant limitation. On the other hand, our approach proved its ability to identify the compounds at the very low levels typical of hair analysis, even when dealing with the potent (i.e., taken at low doses) NSOs. Furthermore, newly discovered NSOs can be added to the panel of target analytes to allow retrospective analysis of previously acquired data to look for the presence of these new substances.

Broad-spectrum HRMS screening methods can become of particular interest owing to the challenges presented by NPS/NSO, and in particular for the continuous modification of drug scenarios in the black market. Furthermore, the retrospective investigation represents an added value for investigation of hair samples; especially when the small amount allows only one analysis, and especially in forensic labs where there is a greater need to maximize the range of detectable compounds. Noteworthy, a screening result always needs a targeted confirmation analysis, especially in forensic cases. However, this is of lesser concern, when the target is already suspected. Indeed, the main challenge with new drugs is the initial identification of candidate drugs for further evaluation. In this scenario, our HRMS-based approach seems very promising and innovative. The only limitation is that the described method still relies on a mass spectral library and an add-on software generating candidate empirical formula. However, with the rapid growth of NPS/NSO, it is likely that HRMS instruments will become increasingly prevalent as forensic screening tools. We envision that our approach can assist national programs of drug surveillance. Indeed, the development of comprehensive screening methods will provide law enforcement agencies and health professionals alike a clearer picture of the long-term use of these drugs and their evolution in the consumer market. Furthermore, the retrospective data analysis feature will become a powerful tool when a new drug is reported for the first time in a certain territory, allowing the monitoring of past consumption trends in specific populations and at different times.

## Electronic supplementary material

ESM 1 (PDF 329 kb).
