# Supplementary material for: Targeted and untargeted detection of fentanyl analogues and their metabolites in hair by means of UHPLC-QTOF-HRMS
Source: Anal Bioanal Chem. 2020 Oct 15;413(1):225–33. doi: 10.1007/s00216-020-02994-x (PMC7801321; doi:10.1007/s00216-020-02994-x)
Supplement: Supplementary file 1 — (PDF 329 kb). [file 216_2020_2994_MOESM1_ESM.pdf]

Analytical and Bioanalytical Chemistry

Electronic Supplementary Material

Targeted and untargeted detection of fentanyl analogues and their metabolites in hair by means of UHPLC-QTOF-HRMS

Alberto Salomone, Daniele Di Corcia, Pierre Negri, Maria Kolia, Eleonora Amante, Enrico Gerace, Marco Vincenti

Table S1 Non-targeted screening of Novel Synthetic Opioids

Compound	Elemental composition	Protonated Exact mass (Da)
Norfentanyl	$C_{14}H_{20}N_2O$	233.1648
Butyrylnorfentanyl	$C_{15}H_{22}N_2O$	247.1805
N-methylnorfentanyl	$C_{15}H_{22}N_2O$	247.1805
4-anilino-benzylpiperidine	$C_{18}H_{22}N_2$	267.1856
Furanylnorfentanyl	$C_{16}H_{18}N_2O_2$	271.1441
4-ANPP	$C_{19}H_{24}N_2$	281.2012
Norcarfentanil	$C_{16}H_{22}N_2O_3$	291.1703
Despropionyl p-fluorofentanyl	$C_{19}H_{23}FN_2$	299.1918
Acetylfentanyl	$C_{21}H_{26}N_2O$	323.2118
Benzylfentanyl	$C_{21}H_{26}N_2O$	323.2118
Methylthiofentanyl	$C_{21}H_{28}N_2O$	325.2274
U-47700	$C_{16}H_{22}Cl_2N_2O$	329.1182
AH-7921	$C_{16}H_{22}Cl_2N_2O$	329.1182
Thienylfentanyl	$C_{19}H_{24}N_2OS$	329.1682
Acrilfentanyl	$C_{22}H_{26}N_2O$	335.2118
Fentanyl	$C_{22}H_{28}N_2O$	337.2274
Methylacetylfentanyl	$C_{22}H_{28}N_2O$	337.2274
Thiofentanyl	$C_{20}H_{26}N_2OS$	343.1839
Cyclopropylfentanyl	$C_{23}H_{28}N_2O$	349.2274
Crotonylfentanyl	$C_{23}H_{28}N_2O$	349.2274
MT-45	$C_{24}H_{32}N_2$	349.2638
Methylfentanyl	$C_{23}H_{30}N_2O$	351.2431
Butyrfentanyl	$C_{23}H_{30}N_2O$	351.2431
Isobutyrylfentanyl	$C_{23}H_{30}N_2O$	351.2431
Hydroxyfentanyl	$C_{22}H_{28}N_2O_2$	353.2224
Methoxyacetylfentanyl	$C_{22}H_{28}N_2O_2$	353.2224
Fluorofentanyl	$C_{22}H_{27}FN_2O$	355.2180
U-49900	$C_{18}H_{26}Cl_2N_2O$	357.1495
Hydroxythiofenatnyl	$C_{20}H_{26}N_2O_2S$	359.1788
Valerylfentanyl	$C_{24}H_{32}N_2O$	365.2587
Methylbutyrilfentanyl	$C_{24}H_{32}N_2O$	365.2587
para-methoxyfentanyl	$C_{23}H_{30}N_2O_2$	367.2380
Methoxyfentanyl	$C_{23}H_{30}N_2O_2$	367.2380
U-50488	$C_{19}H_{26}Cl_2N_2O$	369.1495
4-fluorobutyrfentanyl	$C_{23}H_{29}FN_2O$	369.2337
Fluorobutirylfentanyl	$C_{23}H_{29}FN_2O$	369.2337
Chlorofentanyl	$C_{22}H_{27}ClN_2O$	371.1885
Ocfentanil	$C_{22}H_{27}FN_2O_2$	371.2129
Furanylfentanil	$C_{24}H_{26}N_2O_2$	375.2067
W-15	$C_{19}H_{21}ClN_2O_2S$	377.1085
Cyclopentylfentanyl	$C_{25}H_{32}N_2O$	377.2587
Benzylcarfentanil	$C_{23}H_{28}N_2O_3$	381.2173
Methoxybutyrilfentanyl	$C_{24}H_{32}N_2O_2$	381.2537
Chloroisobutirylfentanyl	$C_{23}H_{29}ClN_2O$	385.2041
Phenylfentanyl	$C_{26}H_{28}N_2O$	385.2274
W-19	$C_{19}H_{22}ClN_3O_2S$	392.1194

Carfentanil	$C_{24}H_{30}N_2O_3$	395.2329
W-18	$C_{19}H_{20}ClN_3O_4S$	422.0936
Benzodioxole fentanyl	$C_{27}H_{28}N_2O_3$	429.2173

Table S2 Intra-day and inter-day accuracy and precision results calculated at 2, 10 and 100 pg/mg, plus recovery and matrix effect for all analytes

	**Accuracy (bias%)**						**Precision (CV%)**						**Matrix effect (±%)**	
	**Intra-day**			**Inter-day**			**Intra-day**			**Inter-day**				
**Analyte**	*Calibration level (pg/mg)*			*Calibration level (pg/mg)*			*Calibration level (pg/mg)*			*Calibration level (pg/mg)*			*Calibration level 2 pg/mg*	
	2	10	100	2	10	100	2	10	100	2	10	100	Mean (±%)	CV%
Norfentanyl	0	9	-16	19	15	13	22	23	12	18	15	12	+17.5	7.5
Acetylfentanyl	6	-14	8	2	-8	-3	25	7	3	24	14	6	-18.4	6.6
Ocfentanil	2	-5	13	1	-7	-3	17	8	4	11	16	10	-58.1	15.2
Acrylfentanyl	4	-10	7	5	-8	0	24	9	5	25	19	13	-59.6	14.9
4-ANPP	3	-3	12	4	-11	2	11	11	3	15	17	12	-18.2	5.4
Fentanyl	6	-11	8	4	-10	-2	24	7	4	19	13	6	-46.1	11.4
Furanylfentanyl	5	-11	8	2	-6	-3	18	8	3	22	12	7	-36.0	10.7
α-Methylfentanyl	5	-9	10	4	-6	-1	19	7	3	21	22	10	-59.6	15.5
Cyclopropylfentanyl	8	-5	5	5	-9	0	18	7	3	17	17	11	-46.2	15.4
Carfentanil	7	-9	2	-5	-10	-1	24	8	4	25	12	9	-42.2	13.9
Butyrfentanyl	4	-3	5	4	-4	-3	18	7	4	24	15	10	-52.1	12.8
4-fluorobutyrfentanyl	17	-2	5	4	-4	-3	12	9	3	17	16	14	-49.0	21.6
